# Reply: The long-term response to imatinib treatment of CML

**DOI:** 10.1038/sj.bjc.6603604

**Published:** 2007-02-06

**Authors:** F Michor

**Affiliations:** 1 Society of Fellows, Harvard University, Cambridge, MA 02138, USA

**Sir**,

Despite the initial success of imatinib (Gleevec, STI571) in the treatment of chronic myeloid leukaemia (CML) (Druker *et al*, 1996), only few patients achieve a complete molecular remission, with just 4% displaying consistent PCR-negative status by 30 months in the IRIS trial (Hughes *et al*, 2003). The source of this residual disease and the question of whether imatinib can deplete leukaemic stem cells have sparked a discussion (Chaudhary *et al*, 1991; Bedi *et al*, 1993; Graham *et al*, 2002; Jiang *et al*, 2004; Michor *et al*, 2005; Roeder *et al*, 2006).

Two groups have recently published theoretical models to study the treatment response to imatinib. Roeder *et al* (2006) investigated stem cell dormancy (Graham *et al*, 2002) as a mechanism of imatinib insensitivity and suggested that proliferating stem cells are depleted during therapy. In the absence of resistance mutations, their model predicts a continuous decrease in the leukaemic cell burden, and eventually an eradication of the disease (Glauche *et al*, 2006). Michor *et al* (2005) proposed that leukaemic stem cells are not depleted by significant amounts during imatinib therapy. This conclusion was drawn from the relapse dynamics in patients who discontinue therapy; their leukaemic cell load increases to levels beyond pretreatment baseline after stop of therapy, suggesting that leukaemic stem cells keep expanding during treatment. We designed the simplest possible model that can reproduce the disease dynamics over the first 12 months of therapy. It was our intent to model CML dynamics over the first year of imatinib therapy only (not over the whole course of disease progression) owing to data availability. The model does not consider competition between wild-type and leukaemic stem cells because there is no evidence for such interactions in the 12 months data. This model predicts an eventual relapse in the leukaemic cell count due to continuously expanding leukaemic stem cells. In a follow-up paper (Dingli and Michor, 2006), we investigated a model including density dependence of wild-type and leukaemic stem cells. In that model, leukaemic stem cells cannot expand indefinitely, but cannot be eradicated either because they are intrinsically insensitive to imatinib. The model reproduces the long-term imatinib response data (Figure 1) and predicts that imatinib cannot cure CML patients.

Our models remain valid given the currently available data. Further experimental and theoretical investigations are needed to increase the understanding of CML stem cell dynamics and to clarify the mechanism of their insensitivity to imatinib.

## Figures and Tables

**Figure 1:**
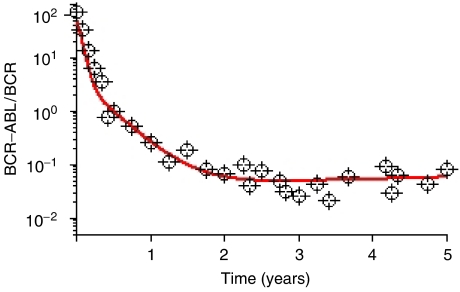
Imatinib treatment dynamics. Based on Dingli and Michor (2006), the system containing stem cells (SC), progenitor cells (PC), differentiated (DC) and terminally differentiated cells (TC) is described by 
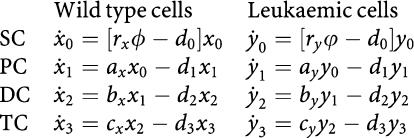
 Here *ϕ*=1/[1+*c*_*x*_(*x*_0_+*y*_0_)] and *ϕ*=1/[1+*c*_*y*_(*x*_0_+*y*_0_)] Imatinib therapy starts on day 0 and leads to a biphasic decline of the leukaemic cell burden. The figure shows the ratio of BCR-ABL over BCR in percent (line) and the median values (circles) of 69 patients from the German cohort of the IRIS trial (Glauche *et al*, 2006). Owing to the unavailability of the raw data, a least squares analysis could not be performed. The leukaemic cell load cannot be eradicated because leukaemic stem cells are insensitive to imatinib. Parameter values are *d*_0_=0.003, *d*_1_=0.008, *d*_2_=0.05, *d*_3_=1, *a*_*x*_=0.8, *b*_*x*_=5, *c*_*x*_=100, *a*_*y*_=3.2 before therapy and *a*_*y*_=0.04 during therapy, *b*_*y*_=5 before therapy and *b*_*y*_=0.167 during therapy, *c*_*y*_=100, *r*_*x*_=0.008, *r*_*y*_=1, *c*_*x*_=1.67 × 10^−6^, *c*_*y*_=3 × 10^−4^.
